# What Attracts People to Visit Community Open Spaces? A Case Study of the Overseas Chinese Town Community in Shenzhen, China

**DOI:** 10.3390/ijerph13070644

**Published:** 2016-06-28

**Authors:** Yiyong Chen, Tao Liu, Xiaohuan Xie, Barbara Goličnik Marušić

**Affiliations:** 1 School of Architecture & Urban Planning, Shenzhen University, Shenzhen 518000, China; chenyiy@szu.edu.cn; 2 Shenzhen Key Laboratory of Built Environment Optimization, Shenzhen University, Shenzhen 518000, China; 3 Center for Population and Development Studies, Renmin University of China, Beijing 100872, China; liutaopku@gmail.com; 4 Urban Planning Institute of the Republic of Slovenia, Ljubljana SI 1000, Slovenia; barbara.golicnik-marusic@uirs.si

**Keywords:** open space, use, community, outdoor activity, Overseas Chinese Town of Shenzhen

## Abstract

A well-designed open space that encourages outdoor activity and social communication is a community asset that could potentially contribute to the health of local residents and social harmony of the community. Numerous factors may influence the use of each single space and may result in a variety of visitors. Compared with previous studies that focused on accessibility, this study highlights the relationship between the utilization and characteristics of community open spaces in China. The Overseas Chinese Town community in Shenzhen is regarded as an example. The association between the number of visitors and space characteristics is examined with multivariate regression models. Results show that large areas with accessible lawns, well-maintained footpaths, seats, commercial facilities, and water landscapes are important characteristics that could increase the use of community open spaces. However, adding green vegetation, sculptures, and landscape accessories in open spaces has limited effects on increasing the outdoor activities of residents. Thus, to increase the use of community open spaces, landscape designers should focus more on creating user-oriented spaces with facilities that encourage active use than on improving ornamental vegetation and accessories.

## 1. Introduction

Public open spaces are key built environment elements within neighborhoods intended to encourage various physical activities, provide a number of significant benefits, and serve various important functions that improve the quality of life in cities [1,2,3]. In urban and landscape planning studies, the quantity and quality of open spaces in a community have been eliciting increasing attention. Open spaces can promote residents’ outdoor activities, which in turn help reduce stress and provide opportunities that promote relaxation [4,5,6,7]. Many dynamic factors and their complex interactions affect the influence of open spaces on human health in urban areas [8]. Based on studies on outdoor activities, many countries have formulated a number of policies and guidelines that guide the planning and design of public open spaces with the aim of promoting the use of open spaces by residents. Landscape design of public open spaces, which usually cover large areas with complex components and functions, have become a challenging task in the field of urban landscape design [9].

A growing body of literature has examined the association between the different aspects of open spaces and physical activity. Kaczynski and Henderson’s literature review indicated that most studies have found that proximity to parks and recreational settings is generally associated with increased physical activity [10]. The spatial configuration of parks, their number, and their accessibility determine their access potential for residential populations [11,12]. The accessibility of open spaces is usually assumed to be the most important factor that influences their use [13,14]. A distance of 300 m to 400 m from a user to an open space is considered an important threshold. When the distance is greater than 400 m, the use frequency decreases rapidly [15]. 

Although the number of studies that focus on accessibility is increasing, several inconsistencies have been found in their results. For example, Lachowycz and Jones’s literature review indicated that only 40% of related studies have confirmed significant associations between access-related measures of local green spaces and physical activity; the majority found weak or no associations [16]. Giles-Corti et al.’s study on the western Australian city of Perth revealed that among open spaces with similar scales, 70% of the respondents opt to visit open spaces that they find most attractive rather than the nearest ones [17]. Schipperijn et al. concluded that distance to green spaces is not a limiting factor for the majority of the Danish population [18]. Nielsen and Hansen also pointed out that only 3% of their respondents consider distance a barrier to the use of public open spaces in Denmark [19]. 

The definition of accessibility needs clarification and deep consideration. The definition of accessibility is based on a gravity model, which is conceived as a measure of the desire and ability of people to overcome distance or travel time to access a facility or activity [17]. Distance from the origin to an open space is still commonly measured with the straight line (Euclidean) distance; however, street-network distance is argued as a better representation of the true relevant spatial distance [11]. A study also suggested the use of pedestrian networks and space syntax to understand the measures of proximity to public open spaces [9]. The representations of open spaces differ in different studies; sometimes, open space entrances are utilized as the destination point [13], whereas commonly, the geographic centroids of public open spaces are used [20]. These different research methodologies and results on accessibility confuse researchers, landscape architects, and policymakers. Thus, creating specific references for landscape design is difficult.

The other factors that influence the use of open spaces are also varied and complicated. The attributes of open spaces per se provide cues about how they are used and by whom. Previous studies have examined the associations between the spatial attributes of open spaces and physical activity or walking. Holman et al. found that the use of parks is influenced by aesthetic features, presence of amenities, and park size [21]. However, Janet and Rachel found that the actual or perceived dimensions of open space size did not directly influence user preferences; instead, adherence to the venue design itself and the venue’s opportunities for activities and landscape appear to be more important [22]. Schipperijn et al. indicated that generally, no association exists between outdoor physical activity and the size of, distance to, and number of features in the nearest urban green space; however, they found positive associations between physical activity and walking/cycling routes, wooded areas, water features, lights, pleasant views, bike rack, and parking [13]. Giles-Corti et al. used a score of open spaces called “attractiveness” to examine the association of open spaces with walking and found that people with good access to attractive open space are 50% more likely to achieve high levels of walking [17]. Goličnik and Ward Thompson reported that having a minimum supplementary space configured to allow for appropriate buffer zones is an important aspect to be considered in public space design and decision making [23]. These research discoveries indicate that physical activity in an urban open space might be stimulated by providing attractive facilities and landscape features. Additionally, the urban open space provision might be brought back to the open space itself. 

Aside from the attributes of open spaces and the distance between users and open spaces, the attributes of users are also important factors that affect the use of open spaces. Studies have found significant variations in the utilization of nearby open spaces by different groups. Among women and men and among younger and older adults, the relationships between activity and living near parks and parklands show significant differences [20]. Age, gender, marital status, and area of residence are listed as important factors that affect patterns in the use of nearby public open spaces [24]. In another study, gender, marital status, age, educational status, and income level together with the reflections of these features were proven to affect user profile at city parks [25]. Several personal factors, including having young children, old age, and poor health, have been proven to be negatively associated with open space use [26]. Preferences for and perceptions of open spaces are influenced not only by users’ social characteristics but also by their recreation activities and specific interests and demands [27].

Existing evidence suggests that users generally prefer proximate, large, and attractive open spaces. Nevertheless, the influence of open space accessibility and scale remains disputable. However, if these disputes could be disregarded and focus is placed on the open space itself, the characteristics that affect the use of open spaces may become clear. What features of open spaces attract users to come and stay in open spaces? Do visitors actually prefer the various facilities inside the space or the attractive environment itself? What can landscape architects do to increase the use of open spaces? These questions still need to be addressed. 

Existing studies have mostly focused on developed cities in Western countries. The rapid urbanization of China in the past 30 years has led to the construction of many public open spaces in high-density communities, but many of them are not user-friendly and thus poorly used [28]. Although Western researchers have provided abundant articles on the use of open spaces, the city structures and characteristics of Western cities are different from those in China. Considering the enormous cultural differences between Eastern and Western societies, research results and recommendations for Western cities are not automatically valid for cities in China [29,30]. Not many studies have investigated the use of public open spaces in China and other Eastern countries. Studies that employed quantitative analyses for open space use are also rare. The differences in user patterns between open spaces in Eastern and Western societies are worth exploring.

The current study regarded the community open space as the specific study subject. On the one hand, community open spaces are usually located inside a residential community, not far from users’ origins (home or work place). Considering that several studies pointed out that distance to a green space (accessibility) is not a limiting factor for most residents [18,19], we focused on other important influencing factors, such as the attractiveness of a space. On the other hand, community open spaces are often small in scale [18,31]. Controlling the influence of the size of the space is possible to some extent. Therefore, we focused on the characteristics of an open space itself. To date, the factors that affect the use patterns of community open spaces are still unclear.

Thus the goals of the current study are twofold: (1) to analyze the specific characteristics of community open spaces that are associated with the use of open spaces in a typical community in China and (2) to explore landscape design strategies through which community open space utilization can be strengthened. 

## 2. Data Acquisition

**Figure 1:**
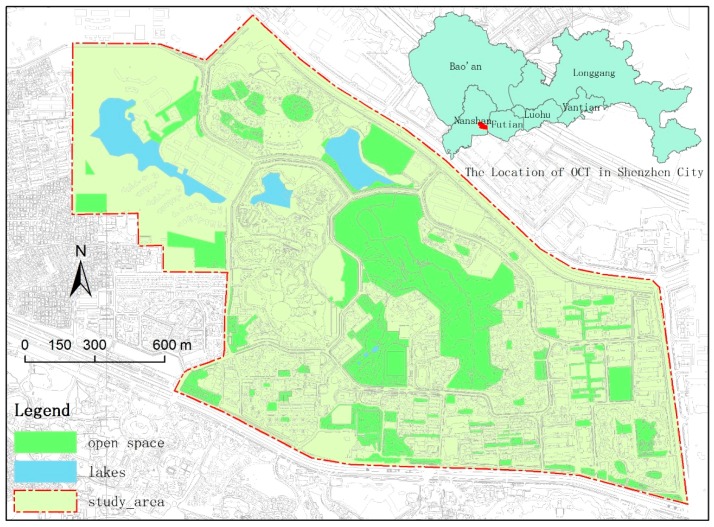
Study area and its location.

This empirical study used the Shenzhen Overseas Chinese Town (OCT) as a case study. We systematically investigated the large communities in the Nanshan District of Shenzhen, which is a pioneering area in China, to look for a mature community with multiple open spaces, good accessibility, and environmental diversity characteristics as a case for our research. The OCT community is located in Nanshan District, Shenzhen City (Figure 1). It is a typical, high-density, mixed community. The entire community is separated from other communities by urban streets in the north, east, and south and by a high wall with an urban village in the west. High-class residential groups are located in the northwest and the southeast, middle-class and low-class residential groups are located in the south and the southwest, and collective dormitory groups exist in the middle. The open space in this community is composed of several community parks, a hilly country park, several lakes, several pedestrian-only commercial zones, and other types of greenery. The distribution of green space in this community is relatively reasonable, and its landscape is ranked as first class in Shenzhen City. The access of residents to open spaces is very high, given that 94.0% of all the residents live within 300 m from an open space [32]. Therefore, this community with high-quality, high-accessibility open spaces is an appropriate case to study the characteristics that influence the use of open spaces.

Before the specific data acquisition process, interviews were conducted with staff members of the OCT administration, the security department, and security guards to obtain a general overview of the community, the management and maintenance of the open spaces, and the public security situation. Through the interviews, we found that the open spaces in the OCT community were planned and developed with high-level standards and are maintained by the OCT Enterprise itself. Therefore, it has high utilization and good public security.

The OCT community was developed and constructed in 1985. It was built and is administrated by a state-owned enterprise, the OCT Group, and has excellent internal facilities and a carefully planned environment [33]. Over the years, the OCT community has become a modernized seashore community that integrates tourism, housing, commercial offices, creative cultural industries, and other functions. According to the staff of the OCT administration, the community’s residential population is 37,700, and the total employed population is approximately 21,800. The community is divided into two parts by Shennan Avenue. The southern part is mainly composed of tourist venues, including Window of the World Theme Park, Folk Culture Village, Splendid China Park, and OCT Happy Bay Entertainment Plaza. The northern part is mainly composed of residential and commercial venues. The northern part of the community, with a total land area of 306.2 ha, was selected as study area in this study.

To establish a theoretical model, three instances of data collection were implemented. First, all the open spaces were identified and then divided into independent units. Second, an environmental scan was conducted to obtain the environmental characteristics of each open space unit. Finally, a systematic four-day observation was carried out to determine the number of users and their activity engagements in each open space unit. 

### 2.1. Open Space Recognition and Unit Division

In 1906, The Open Space Act of Britain provided the first clear definition of open space as follows: “open space” pertains to “any land, whether enclosed or not, on which there are no buildings or of which not more than one-twentieth part is covered with buildings, and the whole or the remainder of which is laid out as a garden or is used for purposes of recreation, or lies waste and unoccupied” [34]. Open space has recently been defined as “managed space, typically green and available and open to all, even if temporally controlled” [35]. In this study, open spaces refer to areas left open for public use in urban communities, whether green or not, such as a plaza, park, playground, or courtyard. The open spaces located in high-class residential communities and villas are not included in this research because they are not open to the general public. In addition, streets, water surfaces, inaccessible hilly areas, and courtyards inside building blocks are excluded. Thus, in this study, open spaces mainly include community parks, outdoor playgrounds, public-access courtyards, water fronts, and small squares around commercial and public buildings.

All open spaces in the OCT community were surveyed, particularly those where visitors can enter and stay to a certain extent. To explore the environmental elements that affect the use of open spaces and control the influence of open space size, large-scale open spaces were divided into space units based on spatial configuration and environmental characteristics. This process was also conducted to maintain the basic conformity or continuity of landscape elements inside the units and simultaneously achieve a large difference or obvious spatial separation between adjacent open space units. Moreover, to guarantee the size comparability of the basic space units, we ensured that the area of each unit is not less than 100 m^2^ and not greater than 20,000 m^2^. The original largest open space in the community is Yanhanshan Hilly Country Park with long wandering walking paths and large woodlands; it was difficult to divide into units. Thus, several user-gathering squares and rest areas were recognized as space units.

According to these principles, all open spaces in the research area were divided into 112 space units with an average area of approximately 2130 m^2^. The composition and functions of each unit differed. Among the 112 space units, 68 were individual land units and 44 belonged to a continuous network of open space or large public parks. Their accessibility had no significant differences [32]; most units were located inside residential or commercial areas, whereas others were located among residential areas. However, the use of the units differed significantly. Several units were always crowded, whereas many others were seldom visited.

### 2.2. Environmental Scanning

Full investigation and documentation of the environmental characteristics of all the space units were conducted. Existing evidence suggests that the characteristics that influence the use of urban public open spaces include the configuration of a place (e.g., park size, walking path, and lawns), facilities (e.g., seats and fitness), and aesthetic features (e.g., water and sculptures) [9,13,36]. Referring to previous studies, the audit content in the current study mainly included three aspects (spatial configuration, facilities, and landscape features) with 17 characteristic variables. For spatial configuration, the total site area (S1), accessible lawn area (S2), woodland area (S3), footpath length (S4), and hard pavement (S5) were measured. The site facilities comprised eight types, namely, fitness facilities (F1), commercial facility sites (located within the open space units and surrounding the open space units, F2), total number of regular seats (F3), auxiliary seats (F4), rain and sun-shading devices (F5), bicycle parking facilities (F6), trash cans (F7), and lighting facilities (F8). The landscape setting surveys included the number of water landscapes (L1), (mammal and bird) biological habitat types (L2), sculptures (L3), and motor vehicle parking volume (L4). Among these variables, S1–S5, F1–F3, and L1–L4 are continuous variables, and F4–F8 are dummy variables.

### 2.3. Observations of Public Open Space Users

Site investigation of the use of all community open space units was conducted in four days in November 2014 (Table 1). During the site investigation, the temperatures fell between 20 °C and 30 °C, and the weather was cloudy or sunny, which is suitable for outdoor activities. A total of 17 trained researchers observed the visitors in the 112 open space units on two weekdays (5 and 13 November) and on two weekends (9 and 16 November). The observation time in each day was from 9 am to 10 pm, and documentation was performed every hour. Each observer was assigned to a residential group, a commercial area, or a park to systematically observe and record the number of visitors to these space units. Each space or space unit was subjected to a 6 min continuous visual scan every hour. Thus, we recorded a sufficient number of visitors, including those who stayed for a long time and those who only stayed for a short period or merely passed by. All visitors to a particular space observed within the 6 min visual scan were recorded as point data on a detailed map (1:250) of the site. 

A total of 35,090 headcounts participating in various outdoor activities (e.g., playing, running, jogging, and reading) were recorded. The average activity population number in each open space unit was 313. During the day (9 am to 6 pm), 27,854 headcounts were observed, and during the evening (7 pm to 10 pm), 7235 headcounts were observed. In total, 14,854 headcounts were observed during weekdays, and 20,236 headcounts were observed during weekends. The number of headcounts utilized in the subsequent text analysis comprises the summary of headcounts observed during the four-day site investigation. 

**Figure 2:**
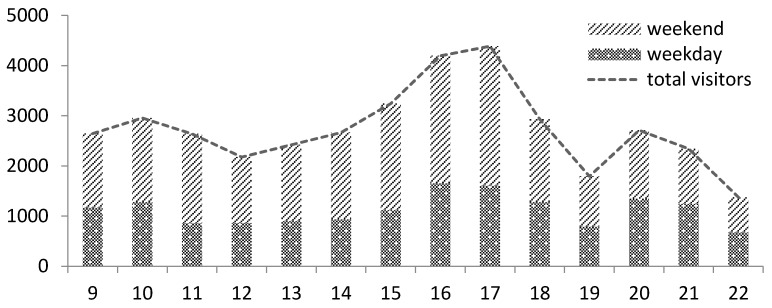
Total visitors observed in all open spaces by hours.

The data confirmed that the open spaces had the most number of visitors from 3 pm to 5 pm; the smallest number was recorded during lunch time and dinner time. In the other time periods within a day, the stream of visitors was relatively stable. After 9 pm, a rapid decrease in the number of visitors was observed (Figure 2).

## 3. Model Building and Results Analysis

### 3.1. Regression Models

In the environment scanning process, 17 variables representing the characteristics of each space unit were scanned. These variables are potential factors that could affect the attractiveness of open spaces and physical activities. However, the initial data analysis showed that several variables should be excluded from the regression model. If the total site area (S1) is introduced to, it would result in a serious multicollinearity problem with several other variables (S2–S5); hence, the model could fail. In addition, the information of S1 could be represented by a summary of S2, S3, and S4. Similarly, the variable hard pavement (S5) contains many trails and has a serious multicollinearity problem with other variables (S1, S4); therefore, it was excluded from the model. For auxiliary seats (F4), which is a dummy variable, the survey indicated that most of the space units provide a certain number of auxiliary seats, such as stairs, lotus pond edges, stones, stone pillars, and sculptures. Thus, the value of F4 in most space units was the same, so we did not introduce F4. For rain and sun-shading devices (F5) and bicycle parking facilities (F6), only several space units provide such facilities. We observed that almost nobody uses them, so we excluded F5 and F6. For trash cans (F7) and lighting facilities (F8), the survey indicated that almost all open spaces provide a sufficient number of these two types of facilities, so they were also excluded.

After the initial selection of influencing factors, seven variables of the observed space units were excluded. The remaining 10 variables were introduced to the regression model, as shown in Equation (1):

(1) $$P_{i}=C+\alpha S_{i}+\beta F_{i}+\gamma L_{i}+\varepsilon_{i}$$

where $P_{i}$ represents the total activity in open space unit *i*; *C* is a constant term; ε*_i_* is the disturbance; and $S_{i}$, $F_{i}$, and $L_{i}$ represent the spatial composition, facilities, and environmental elements of the open space units, respectively. The basic information of the 10 introduced independent variables and the dependent variable is shown in Table 2. The model illustrates the influencing factors of the total activity ($P_{i}$) of open space unit *i*. Three regressions were completed, namely, total, weekdays, and weekends, because the activity patterns during weekdays and weekends are somewhat different.

First, single-factor models were established to fit the three variable groups (model 1). Second, a multi-factor model was established to estimate the comprehensive influence of these variables (model 2). Third, weekday and weekend multi-factor models were established to test the different influences of the variables during weekdays and weekends. The regression results are shown in Table 3.

To investigate the influence of existing (or not existing) facilities (F) and environmental elements (L) on the use of open spaces, we established a dummy variable model (model 3), in which all the variables of facilities (F) and environmental elements (L) are transformed into dummy variables. If a certain type of facility (F) or environmental element (L) exists in one open space unit, then the value of this variable is 1; otherwise, the value is 0. The regression results are presented in Table 4.

### 3.2. Results

In the regression models, the correlation among the independent variables was less than 0.5, and VIF was less than 2. No evident collinearity problems were observed [37,38]. The fitting result of the single-factor model revealed that two variables of each type significantly affect the use frequency of open spaces (Table 3). The symbols and significance of the variables of the estimated coefficient were consistent with those in the multi-factor model. This consistency further verifies the stability of the models. Meanwhile, the attractiveness-promotion effect of the reasonable configuration of spatial resources (spatial configuration) was significantly higher than that of site facilities and environmental elements. The goodness-of-fit value of the former was 0.218, and the separate explanatory powers of the latter were less than 0.1. The goodness-of-fit values of the multi-factor model (model 2) and the dummy variable model (model 3) were 0.388 and 0.389, respectively (approximately 40%).

#### 3.2.1. Influence of Spatial Configuration

Space is the carrier of public activities. Among the different types of spaces, paved footpath (S4), which had a high utilization rate, was the activity space of residents and the connection channel of different spaces. The regression results indicated that when the length of footpath increases by 10 m, the number of visitors in this area is expected to increase by seven. The influences of weekdays and weekends slightly differed (Table 3).

**Figure 3:**
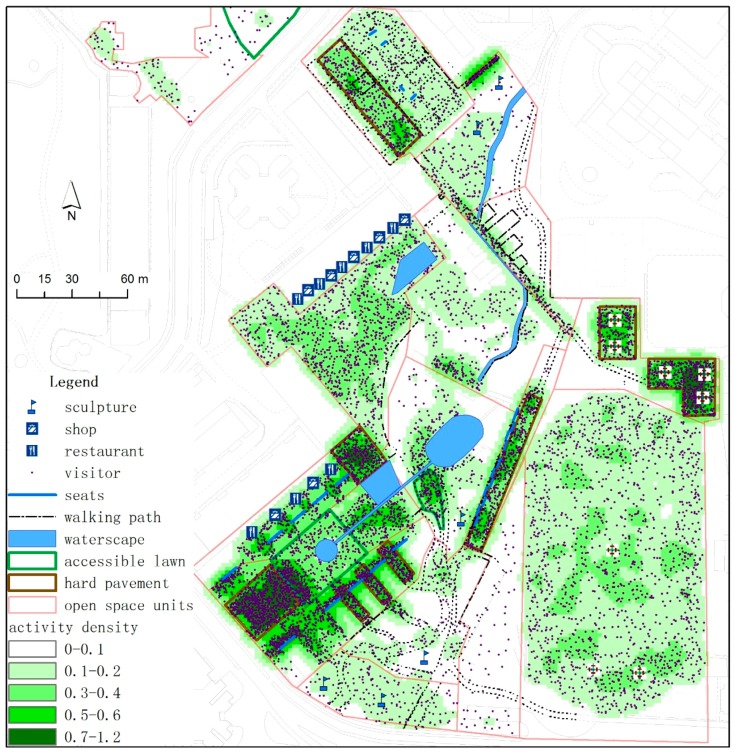
Map showing the activity density in the Ecological Square Park.

The accessible lawn area (S2), which is important in attracting visitors, provided an open view and great accessibility. When the lawn area increases by 100 m^2^, the number of visitors in this area is expected to increase by nine and four during weekdays and weekends, respectively. The density distribution of total visitor flow during weekdays and weekends showed that accessible lawn and hard pavement are characterized by a large flow density (Figure 3). These spaces are also considered the main public areas for visitors to play and stay in.

**Figure 4:**
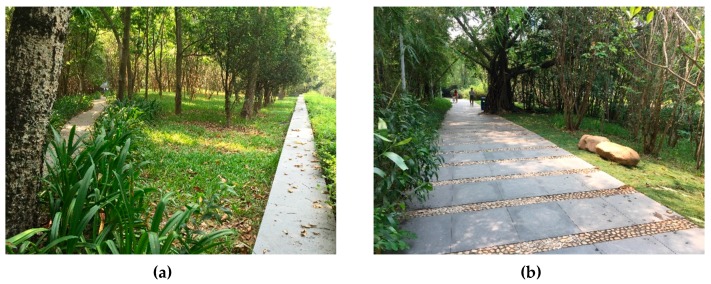
Photo showing a walking path crossing densely covered woodlands. (**a**) Narrow walking path without visitors; (**b**) Wide walking path with few visitors.

Contrary to general understanding, our findings indicate that in the community, the woodland (S3) is not significantly associated with the number of users. The model revealed its weak exclusion effect on open space users. The investigation showed that woodlands occupied much space, and most woodland open spaces lack a carefully landscaped design. In several observed space units, woodlands occupied over 80% of the total area, leaving little active space for users. Dense woods also cause safety problems at night. Several crimes have been reported in these densely covered woodlands according to our interviews. In the Ecological Square, the overall visitor flow was large, but the dense woodland areas were inaccessible (Figure 3 and Figure 4). The units with dense vegetation were equipped with several walkways only; the lack of activity and places to stay leads to a low visitor flow and low activity density.

#### 3.2.2. Influence of Facilities

**Figure 5:**
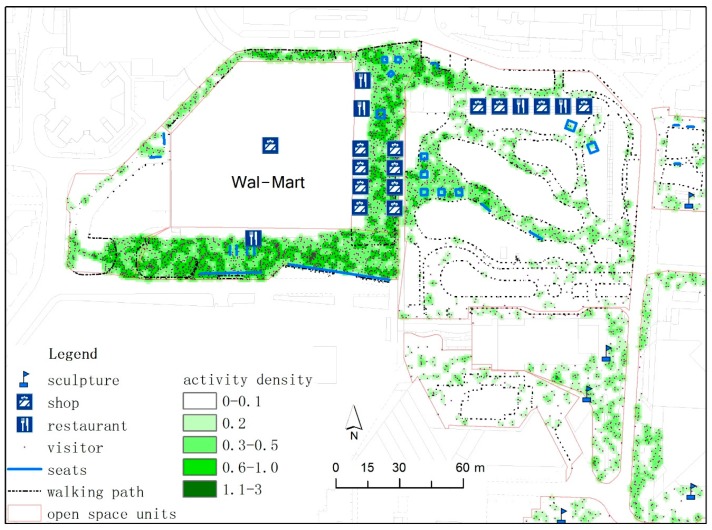
Map showing the activity density around Wal-Mart supermarket.

Site facilities significantly influenced the improvement of site attractiveness and the use of the open spaces. Various commercial service facilities (F2) were found to be important in attracting visitors to the open spaces. The regression model showed that the addition of one commercial facility is expected to attract two and eight additional visitors to play in an area on weekdays and weekends, respectively. This trend indicates that commercial consumption occurs more remarkably on weekends than on weekdays. Likewise, visitors stay longer in commercial facilities on weekends than on weekdays. For example, our field investigation (Figure 5) showed that the total activity density during weekdays and weekends surrounding the commercial facilities of the OCT Wal-Mart was five times higher than that of the surrounding areas. As the distance from Wal-Mart increases, the activity density decreases sharply.

The number of benches (F3) in an area also influenced the decision of visitors to stay. Each additional bench is expected to attract five additional visitors to play in that area. This effect was almost similar during weekdays and weekends. Considering that weekends had more visitors than weekdays, we estimate that benches are probably intensely used and that additional seats should be provided. The field observations during the weekend showed that even in the space units with the most number of benches, all the benches are almost always occupied. Several older users have to sit on auxiliary seats because all benches are full. The strong guiding effect of benches on attracting visitors corresponds to their scarcity. If additional benches are added to the right places, then visitors would be encouraged to stay, which would enhance the attractiveness of the space for community residents, urge residents to participate in leisure, exchange, and other community activities, and strengthen community cohesion [39]. 

In contrast to the general concepts regarding the role of fitness facilities in urban open spaces, our actual results showed that in community open spaces, the availability of fitness facilities (F1) does not certainly attract more visitors. Not all facilities are fully used; regardless of the number of established fitness facilities in different space units, the number of users is almost the same. Increasing the number of fitness facilities does not attract more visitors to the space. In model 3, whether the supply of fitness facilities in the observed space unit significantly influences visitor flow is discussed.

#### 3.2.3. Influence of Ground Environment Elements

The number of water landscapes (L1) remarkably affected the number of visitors. Each increase in water landscapes is expected to attract 73 and 88 visitors to stay on weekdays and weekends, respectively. This trend reflects the strong preference of visitors for water landscapes. This preference is also a driving force for real estate businesses to construct community water landscapes. Water landscapes in OCT mainly include lakes, pools, fountains, and streams. The observations revealed a large visitor flow in almost all the surrounding water landscapes. The diverse activities include fishing, swimming, photographing, and sightseeing. 

Contrary to general understanding, our observation and regression indicate that there is no significant relationship between the number of sculptures (L3) and the activity density. The sculptures were set as accessories of the place, usually occupied the limited activity space, and were not integrated into users’ activities. Tourists may come to have a glance at the sculptures or take photographs, but they leave immediately afterward. There are very few local residents opt to stay near the sculptures. No evident increase in visitor flow was observed around the sculptures. Therefore, placing more sculptures in the community open space will not attract more users.

Animal species (L2) is usually a factor that attracts users, but the regression model revealed great exclusion between animals and crowd activities. In this observation, the term “animals” refers to wild animals instead of pets; these animals mainly include birds and mammals that live in quiet habitats. The number of activities in an open space also presented exclusion to the number of parking spaces (L4). On the one hand, parking occupied the outdoor activity fields; on the other hand, vehicles were not attractive to visitors. The regression result was consistent with our expected finding, but it was not significant.

#### 3.2.4. Comparison of Regression Models

Comparison of the dummy-variable model (model 3), the multifactor model (model 2), and the single-factor model (model 1) revealed that the effects of most variables remained stable; this finding indicates model stability. Several differences were also found in the models.

First, in the comparison of models 2 and 3, although the influence of fitness facilities (F1) was still not significant, the *t* value increased from 0.16 to 1.1 in model 3. Therefore, the installation of fitness facilities is highly essential, but the number of facilities inside a space unit is unimportant. Thus, the spatial layout of fitness facilities should be relatively dispersed instead of concentrated in community open spaces. 

Second, the significance of commercial facilities improved significantly in model 3. Therefore, the number of commercial facilities is relevant, but the presence or absence of commercial facilities is more important than the number of commercial facilities. As such, the layout of commercial facilities should be a combination of concentration and dispersion. This finding implies that large commercial facilities and numerous dispersed small commercial locations in the community are beneficial. 

Third, the presence or absence of benches showed insignificant effects (model 3), but numerous benches were more attractive to visitors (model 2) than a few benches. Hence, benches or seats have a large demand. Increasing this kind of facility would attract more visitors, whereas individual facilities provide a limited influence. The layout of seats should be a combination of concentration and dispersion, similar to that of commercial facilities. In particular, service object, entrance distribution, and leisure path should be integrated into the pre-phase analysis to predict the spatial distribution of visitor flow and activities and determine the areas that should be equipped with seats. 

Finally, the exclusion of visitors from a parking lot (existing or not) was evident; this exclusion was slightly related to the scale and number of parking spaces. Having a parking space in an open space is not advisable; relevant measures should be implemented to prohibit vehicles from entering open spaces. If this situation cannot be avoided, then the best way is to place the parking lot at a relatively concentrated place, such as important individual entrances, to reduce its negative effects on the quality and attractiveness of open spaces. 

## 4. Discussion

### 4.1. Discussion on the Influencing Factors of Community Open Spaces

Some community open spaces are always crowded, whereas others have a few visitors. Traditional research on this topic in relation to accessibility has shown remarkable progress. The present study confirms that the landscape characteristics of open spaces are also important influencing factors. As concluded by Handy and Niemeier’s study on accessibility, the quality and use of open spaces should be given special attention [40]. We found significant correlations between the use of green spaces and the characteristics of open spaces in our study.

Scholars have been presenting questions regarding the attractiveness of open spaces since the last century. For instance, do visitors always prefer large-scale open spaces? Holman et al. argued that proximity and accessibility are important, but they alone do not explain patterns of use; many other factors, including environmental aesthetics, animals, maintenance, space scale, and footpath length, influence the use of open spaces [21]. Talbot and Kaplan pointed out that real and perceived scales are not directly related to users’ preferences; conversely, site adhesion, opportunities, and landscape are essential [22]. Ye et al. indicated that in terms of daily leisure activities in urban open spaces, the quality of a “micro environment (local environment)” is more beneficial than that of the overall environment [41]. Various peripheral factors, including the attributes of users, social psychology environment, surrounding facilities, and supply–demand relationship, may also affect the use of open spaces [17]. The current study further confirms that accessible lawn area, footpath length, benches, commercial facilities, and other factors are important characteristics that affect the use of open spaces.

Many factors influence the use of open spaces, and these mechanisms remain unclear. Selecting among different open spaces with different characteristics is important because different open spaces complement one another [26]. When asked about the factors that they liked about the open spaces [42] or factors that influence their use of these spaces for physical activity [21] through questionnaires, the respondents mentioned trees, water features, bird life, and activity space. This scenario is consistent with our correlation analysis, except for trees and bird life, and the possible reasons are discussed in Section 3.2. Kaczynski et al. found that park facilities, including paved trails, water area, and playground, are more important than park amenities, such as drinking fountain, picnic area, and restroom for physical activity [43]. Our study also found a significant relationship between the use of open spaces and walking path, water landscapes, and accessible lawn; no significant relationship was found between the use of open spaces and open space amenities, such as excess decorations, heavy woodlands, sculptures, and fitness facilities. Schipperijn et al. found positive associations between physical activity and size, walking or cycling routes, wooded areas, water features, lights, pleasant views, bike rack, and parking lot of urban green space [13]. These results are mostly consistent with those of our study, except for some inconsistencies. Considering that our study focused on community open spaces that are small in scale in high-density residential areas in China, the characteristics that affect use patterns differ in open spaces of different types with distinctive characteristics.

Numerous characteristics of open spaces may influence their use. Thus, in urban and landscape design, the numerous features of open spaces should be considered. Many Western countries have formulated a number of specific policies and guidelines that oversee open space planning and landscape design. However, in Eastern societies such as China, detailed policies and guidelines on the supply of open spaces are still absent, with only a few simple standards. For example, according to the national code for the classification of urban land use and planning standards, the planned urban green space per capita should not be less than 10.0 m^2^, of which the green land area per capita should not be less than 8.0 m^2^. In this code, the supply standard for required facilities and landscape amenities is absent [44]. Thus, many open spaces are characterized by a single type, a monotonous function, scarce facilities, and a low utilization rate. Meanwhile, the construction of city parks remarkably considers the quantity requirement of greening coverage. For example, the Shenzhen Urban Planning Standards and Guidelines [45] recommend that the greening rate of a park should not be less than 65%, and the width of the green belt in the community park should not be less than 8 m. Therefore, the construction of parks remarkably considers greening; these parks unilaterally emphasize landscaping, gardening, and ornamental features with lush trees and rich vegetation. As a result, the activity space for users is limited or absent. This study suggests that the most attractive factors of open spaces to users are accessible lawn area, footpath length, and site facilities, not woodland, flowers, plants, or greening coverage. These attractive factors are directly related to the demand of users. In terms of the supply of open spaces, the Hong Kong Planning Standards and Guidelines provide the per capita indicator of open spaces and recommend a passive open space-to-active open space ratio of not less than 3:2 [46]. This recommendation ensures the demand of sufficient land for all kinds of dynamic activities and serves as a helpful reference.

### 4.2. Implications for Landscape Architecture

Well-designed open spaces are an important community asset because they serve as a venue for outdoor physical activities and social interaction. In open spaces in high-density residential communities, attracting more users is possible by providing more accessible mown lawns, developing more well-maintained footpaths, providing more seats and commercial facilities, and creating water landscapes. Encouraging the increased use of public spaces would have a positive effect on attracting users to public spaces, thereby increasing the security of public open spaces [47].

Redesigning existing spaces is also important for the promotion of public open spaces in communities [17]. Redesigning open spaces by adding accessible fields for users, including accessible lawns, hard-covered fields, and footpaths for multiple users—walkers, joggers, cyclists, and passive recreational users (e.g., sitters, observers)—is highly recommended. In this manner, an important community resource is maximized. 

Open spaces are of different types. For example, in the UK, open spaces are classified into nine categories, namely, parks and ornamental gardens, natural and semi-natural green spaces, green corridors, outdoor sports facilities, amenity green spaces, provision for children and young people, allotments, cemeteries, and civic spaces [48]. The provision and distribution of different categories depend on the function of the spaces. The primary function of community open spaces is to provide predominantly passive recreation and sitting areas and children’s playgrounds to serve local residents close to home; these spaces are passive in nature and positive in providing a venue for resting [46]. The provision of community open spaces is based on the number of residents. Thus, community open spaces are usually much smaller than urban and regional open spaces. Small urban parks should be designed with natural components, shielded from disturbing surroundings, and furnished with some seating to promote opportunities for restorative experiences and to function as social meeting places [49]. This study also showed that open space units providing facilities, activity spaces, and amenity landscape features are more welcoming to visitors. Therefore, the landscape design of open spaces in high-density residential communities should be concordant with the function of such spaces, that is, providing active spaces for local residents.

Excessive ornamental vegetation, sculptures, and dense woodlands are not recommended for community open spaces. In some community parks in China, the greening coverage occupies more than 65% of the public space [50], leaving little actual open space for visitors. Providing sufficient regular and various kinds of auxiliary seats is also important [51]. The total length density of seats in community open spaces in Hong Kong is about 10 m/100 m^2^, but in the OCT community, the total length density of seats is less than 3 m/100 m^2^. Fitness facilities are also essential for communities, including fitness facilities for the elderly, children’s entertainment facilities, table tennis tables, badminton fields, and the like. 

## 5. Conclusions and Future Studies

This study confirms that large accessible lawns, well-maintained footpaths, seats, commercial facilities, and water landscapes are important characteristics that could increase the use of community open spaces. It argues that increasing the green vegetation and the number of sculptures or landscape accessories in community open spaces exerts limited effects on residents’ outdoor activities. Too much woodland and excessively large green coverage do not significantly influence the residents’ outdoor activities in community open spaces. Sculptures or sketches serve as decorations and do not encourage citizens to stay. Thus, to increase the use of community open spaces, landscape designers should focus on creating user-oriented spaces with facilities that encourage active use instead of spending excessive money on ornamental vegetation and accessories. 

This study focused on the characteristics and utilization of community open spaces; the characteristics of users were not recorded. The characteristics of users, including age, gender, and group, may also affect the use of community open spaces. In addition, the characteristics of an open space that attract one population group may also affect its use by other groups. 

Given the limitation of the observation method, we could only record the headcounts of visitors who appeared in a certain open space. We could not distinguish the headcounts of those who stayed for a long period (e.g., for more than half an hour) and the headcounts of those who only stayed for several minutes or were merely passing by. These differences may also be associated with the landscape characteristics of an open space. Studies could be conducted after systematic questionnaires have been distributed and investigations have been conducted. With this recommendation in mind, future studies can explore other types of public open spaces after comparing the characteristics of different open spaces and utilization across different groups. Comparative studies could also be conducted after performing additional case studies.

## Figures and Tables

**Table 1:** Information of variables in the environmental scanning.

Type	Variables	Symbol	Definition	Measurement
Spatial configuration	total site area	*S1*	land area of the site	m^2^
	accessible lawn area	*S2*	lawn that visitors could enter and stay in	m^2^
	woodland area	*S3*	woodland area that visitors could enter and stay in	m^2^
	footpath length	*S4*	length of walking path, green way, and pedestrian line	m
	hard pavement	*S5*	hard covered floor area, usually a small plaza or square	m^2^
Facilities	fitness	*F1*	outdoor fitness facilities, such as the bars, and fitness facilities for the elderly	count
	commercial	*F2*	commercial facilities located within the open space units and surrounding the open space units	count
	regular seats	*F3*	regular seats, such as seats and benches	count
	auxiliary seats	*F4*	irregular seats but could be sat on, such as flower bed edges, stairs, lotus pond edges, stone pillars, and sculptures	yes/no
	rain and sun-shading devices	*F5*	rain and sun-shading cover, including shadow under building roof	yes/no
	bicycle parking facilities	*F6*	bicycle parking ground and bicycle stands	yes/no
	trash cans	*F7*	dustbins and trash cans	yes/no
	lighting facilities	*F8*	any kind of lighting	yes/no
Landscape features	water landscapes	*L1*	water-related landscapes, such as lakes, pools, fountains, and streams	count
	biological habitat types	*L2*	mammal and bird biological habitat types, not the number of animals	count
	sculptures	*L3*	all kinds of sculptures and street furniture used for decoration	count
	motor vehicle parking on site	*L4*	ground parking spaces, including illegal parking	count

**Table 2:** Basic information of all variables involved.

Type	Variables	Symbol	Mean Value	Standard Deviation	Minimum	Maximum
Total activity	sum (count)	*P*	312.089	378.504	3	2101
	weekday (count)	*P1*	131.964	166.321	0	846
	weekend (count)	*P2*	180.125	228.576	1	1255
Spatial configuration	accessible lawn area (m^2^)	*S2*	162.991	812.542	0	8000
	woodland area (m^2^)	*S3*	140.732	242.446	0	2400
	footpath length (m)	*S4*	153.973	165.103	0	1000
Facilities	fitness (count)	*F1*	0.554	2.423	0	20
	commercial (count)	*F2*	2.545	5.238	0	40
	seats (count)	*F3*	2.839	6.946	0	50
Landscape features	water landscape (count)	*L1*	0.348	0.694	0	2
	biological habitat types (count)	*L2*	0.277	0.557	0	2
	sculptures (count)	*L3*	0.286	0.544	0	2
	motor vehicle parking (count)	*L4*	2.795	6.503	0	30

**Table 3:** Single-factor and multi-factor regression models.

Variables	Single-Factor Model (Model 1)			Multi-Factor Model (Model 2)		
	Spatial Configuration	Facilities	Landscape Features	Total	Weekday	Weekend
Accessible lawn area (S2)	0.14 ***			0.14 ***	0.04 **	0.09 ***
	(3.16)			(3.07)	(2.15)	(3.44)
Woodland area (S3)	−0.14			−0.13	−0.02	−0.11
	(−1.02)			(−1.01)	(−0.42)	(−1.34)
Footpath length (S4)	0.60 ***			0.67 ***	0.32 ***	0.34 **
	(2.79)			(2.90)	(3.10)	(2.44)
Fitness (F1)		−4.69		1.99	1.09	0.90
		(−0.32)		(0.16)	(0.19)	(0.12)
Commercial (F2)		16.62 **		9.67	1.61	8.06 **
		(2.49)		(1.58)	(0.58)	(2.16)
Seats (F3)		9.33 *		10.14 **	5.12 **	5.01 *
		(1.85)		(2.20)	(2.45)	(1.79)
Water landscape (L1)			173.59 ***	162.22 ***	73.34 ***	88.88 ***
			(2.77)	(2.98)	(2.96)	(2.68)
Biological habitat types (L2)			−201.67 **	−270.58 ***	−127.50 ***	−143.08 ***
			(−2.61)	(−3.72)	(−3.86)	(−3.23)
Sculptures (L3)			−0.24	−9.08	−7.35	−1.73
			(−0.00)	(−0.16)	(−0.28)	(−0.05)
Motor vehicle parking (L4)			−6.28	−6.39	−3.30	−3.09
			(−1.14)	(−1.27)	(−1.44)	(−1.01)
Cons	216.03 ***	245.92 ***	325.08 ***	189.75 ***	80.24 ***	109.52 ***
	(4.55)	(5.92)	(6.88)	(3.85)	(3.58)	(3.65)
*N*	112	112	112	112	112	112
*R*2	0.218	0.086	0.092	0.388	0.344	0.377
adj. *R*2	0.196	0.061	0.058	0.328	0.279	0.316

The values in brackets are *t* values; * *p* < 0.1, ** *p* < 0.05, *** *p* < 0.01.

**Table 4:** Dummy-variable model (model 3).

Variables	Total	Weekdays	Weekends
Accessible lawn area (S2)	0.12 ***	0.04 *	0.08 ***
	(2.70)	(1.96)	(2.99)
Woodland area (S3)	−0.16	−0.03	−0.13
	(−1.22)	(−0.53)	(−1.61)
Footpath length (S4)	0.64 ***	0.28 ***	0.36 ***
	(3.01)	(2.86)	(2.81)
Fitness (F1)	129.21	37.88	91.34
	(1.10)	(0.71)	(1.29)
Commercial (F2)	163.13 **	46.80 *	116.43 ***
	(2.62)	(1.65)	(3.10)
Seats (F3)	97.70	70.50 **	27.20
	(1.48)	(2.33)	(0.68)
Water landscapes (L1)	206.45 **	88.45 **	118.00 **
	(2.41)	(2.26)	(2.28)
Biological habitat types (L2)	−297.04 ***	−132.47 ***	−164.57 ***
	(−3.26)	(−3.18)	(−2.99)
Sculptures (L3)	−17.90	−14.79	−3.11
	(−0.24)	(−0.44)	(−0.07)
Motor vehicle parking (L4)	−153.07 **	−59.35 *	−93.71 **
	(−2.03)	(−1.72)	(−2.06)
Cons	161.64 ***	67.28 **	94.36 **
	(2.66)	(2.42)	(2.57)
*N*	112	112	112
*R*2	0.389	0.339	0.389
adj. *R*2	0.328	0.273	0.329

The values in brackets are *t* values; * *p* < 0.1, ** *p* < 0.05, *** *p* < 0.01.

## References

[1] Kaczynski A.T., Henderson K.A. (2008). Parks and recreation settings and active living: A review of associations with physical activity function and intensity. J. Phys. Act. Health.

[2] Coutts C., Chapin T., Horner M., Taylor C. (2013). County-level effects of green space access on physical activity. J. Phys. Act. Health.

[3] Bowler D.E., Buyung-Ali L., Knight T.M., Pullin A.S. (2010). Urban greening to cool towns and cities: A systematic review of the empirical evidence. Landsc. Urban Plan..

[4] Grahn P., Stigsdotter U.A. (2003). Landscape planning and stress. Urban For. Urban Green..

[5] Jansson M., Fors H., Lindgren T., Wiström B. (2013). Perceived personal safety in relation to urban woodland vegetation—A review. Urban For. Urban Green..

[6] Roe J.J., Ward Thompson C., Aspinall P.A., Brewer M.J., Duff E.I., David M., Richard M., Angela C. (2013). Green space and stress: Evidence from cortisol measures in deprived urban communities. Int. J. Environ. Res. Public Health.

[7] Jiang B., Chang C.Y., Sullivan W.C. (2014). A dose of nature: Tree cover, stress reduction, and gender differences. Landsc. Urban Plan..

[8] Tzoulas K., Korpela K., Venn S., Yli-Pelkonen V., Kaźmierczak A., Niemela J., James P. (2007). Promoting ecosystem and human health in urban areas using Green Infrastructure: A literature review. Landsc. Urban Plan..

[9] Koohsari M.J., Mavoa S., Villanueva K., Sugiyama T., Badland H., Kaczynski A.T., Owen N., Giles-Corti B. (2015). Public open space, physical activity, urban design and public health: Concepts, methods and research agenda. Health Place.

[10] Kaczynski A.T., Henderson K.A. (2007). Environmental correlates of physical activity: A review of evidence about parks and recreation. Leis. Sci..

[11] Zhang X., Hua L., Holt J.B. (2011). Modeling spatial accessibility to parks: A national study. Int. J. Health Geogr..

[12] Koohsari M.J., Kaczynski A.T., Giles-Corti B., Karakiewicz J.A. (2013). Effects of access to public open spaces on walking: Is proximity enough?. Landsc. Urban Plan..

[13] Schipperijn J., Bentsen P., Troelsen J., Toftager M., Stigsdotter U.K. (2013). Associations between physical activity and characteristics of urban green space. Urban For. Urban Green..

[14] Barbosa O., Tratalos J.A., Armsworth P.R., Davies R.G., Fuller R.A., Johnson P., Gaston K.J. (2007). Who benefits from access to green space? A case study from Sheffield, UK. Landsc. Urban Plan..

[15] Badland H., Hickey S., Bull F., Giles-Corti B. (2014). Public transport access and availability in the RESIDE study: Is it taking us where we want to go?. J. Transp. Health.

[16] Lachowycz K., Jones A.P. (2011). Greenspace and obesity: A systematic review of the evidence. Obes. Rev. Off. J. Int. Assoc. Study Obes..

[17] Giles-Corti B., Broomhall M.H., Knuiman M., Collins C., Douglas K., Ng K., Lange A., Donovan R.J. (2005). Increasing walking: How important is distance to, attractiveness, and size of public open space?. Am. J. Prev. Med..

[18] Schipperijn J., Ekholm O., Stigsdotter U.K., Toftager M., Bentsen P., Kamper-Jørgensen F., Randrup T.B. (2010). Factors influencing the use of green space: Results from a Danish national representative survey. Landsc. Urban Plan..

[19] Nielsen T.S., Hansen K.B. (2006). Nearby nature and green areas encourage outdoor activities and decrease mental stress. Cab Rev. Perspect. Agric. Vet. Sci. Nutr. Nat. Res..

[20] Kaczynski A.T., Potwarka L.R. (2009). Association of parkland proximity with neighborhood and park-based physical activity: Variations by gender and age. Leis. Sci..

[21] Holman C., Donovan R., Corti B. (1996). Factors influencing the use of physical activity facilities: Results from qualitative research. Health Promot. J. Austr..

[22] Janet Frey T., Rachel K. (1986). Judging the sizes of urban open areas: Is bigger always better. Landsc. J..

[23] Goličnik B., Ward Thompson C. (2010). Emerging relationships between design and use of urban park spaces. Landsc. Urban Plan..

[24] Sanesi G., Chiarello F. (2006). Residents and urban green spaces: The case of Bari. Urban For. Urban Green..

[25] Yilmaz S., Zengin M., Yildiz N.D. (2007). Determination of user profile at city parks: A sample from Turkey. Build. Environ..

[26] Schipperijn J., Stigsdotter U.K., Randrup T.B., Troelsen J. (2010). Influences on the use of urban green space—A case study in Odense, Denmark. Urban For. Urban Green..

[27] Roovers P., Hermy M., Gulinck H. (1998). Visitor profile, perceptions and expectations in forests from a gradient of increasing urbanisation in central Belgium. Landsc. Urban Plan..

[28] Monda K.L., Penny G.L., June S., Popkin B.M. (2007). China’s transition: The effect of rapid urbanization on adult occupational physical activity. Soc. Sci. Med..

[29] Fatemi N. Urban green space in a high-density city: User expectations, accessibility and experience in context of Dhaka. Proceedings of the International Conference on Cities, People and Places-Iccpp.

[30] Ma B.C., Sang Q., Gou J.F. (2014). Shading effect on outdoor thermal comfort in high-density city: A case based study of Beijing. Adv. Mater. Res..

[31] Baur J.W.R., Tynon J.F. (2010). Small-scale urban nature parks: Why should we care?. Leis. Sci..

[32] Chen Y. (2016). Influential factors of the amount of community open space activity. J. Shenzhen Univ. Sci. Eng..

[33] Liu J. (2007). Exploration of public space developed and managed by enterprise—Case study of the Huaqiaocheng Ecological Square pattern. Chin. Landsc. Archit..

[34] Open Space Act. http://www.unesco.org/culture/natlaws/media/pdf/gb/uk_openspacesact_enorof.

[35] Carmona M. (2010). Contemporary public space, part Two: Classification. J. Urban Des..

[36] Gobster P.H. (2002). Managing urban parks for a racially and ethnically diverse clientele. Leis. Sci..

[37] Belsley D.A., Kuh E., Welsch R.E. (1980). Regression diagnostics—Identifying influential data and sources of collinearity. J. Mark. Res..

[38] O’Brien R.M. (2007). A caution regarding rules of thumb for variance inflation factors. Qual. Quant..

[39] Gehl J. (2010). Cities for People.

[40] Handy S.L., Niemeier D.A. (1997). Measuring accessibility: An exploration of issues and alternatives. Environ. Plan. A.

[41] Ye P., Wang H., Gao F. (2012). A preliminary study on the research method of urban public space environment and behavior based on GPS: A case study of Shengli Square in Hefei. Archit. J..

[42] Tinsley H.E.A., Tinsley D.J., Croskeys C.E. (2002). Park usage, social milieu, and psychosocial benefits of park use reported by older urban park users from four ethnic groups. Leis. Sci..

[43] Kaczynski A.T., Potwarka L.R., Saelens B.E. (2008). Association of park size, distance, and features with physical activity in neighborhood parks. Am. J. Pub. Health.

[44] Ministry of Housing and Urban-Rural Development (MHURD) (2011). Code for Classification of Urban Land Use and Planning Standards of Development Land (GB50137–2011).

[45] Municipal People’s Government of Shenzhen (MPGS) (2013). Shenzhen Urban Planning Standards and Guidelines.

[46] Urban Planning Committee of Hong Kong (UPCHK) (2014). Hong Kong Planning Standards and Guidelines.

[47] Kuo F.E., Sullivan W.C. (2001). Environment and crime in the inner city: Does vegetation reduce crime?. Environ. Behav..

[48] Urban Green Spaces Taskforce (UGST) (2002). Green Spaces, Better Places: The Final Report of the Urban Green Spaces Taskforce.

[49] Nordh H., Østby K. (2013). Pocket parks for people—A study of park design and use. Urban For. Urban Green..

[50] Ministry of Construction of China (2002). Standard for Classification of Urban Green Space.

[51] Chen Y., Liu T., Liu W. (2016). Increasing the use of large-scale public open spaces: A case study of the North Central Axis Square in Shenzhen, China. Habitat Int..

